# Risk factors, treatment outcomes and predictors of death of newly diagnosed patients with venous thromboembolism in a quaternary hospital in Ghana

**DOI:** 10.4314/gmj.v59i4.3

**Published:** 2025-12

**Authors:** Amoako Duah, Yvonne A Nartey, Kwame Ekremet

**Affiliations:** 1 Department of Medicine, University of Ghana Medical Centre Ltd, Legon, Ghana; 2 Department of Internal Medicine, School of Medical Sciences, University of Cape Coast, Ghana; 3 Department of Emergency Medicine, University of Ghana Medical Centre Ltd, Legon, Ghana

**Keywords:** Venous thromboembolism, Risk factors, Treatment outcomes, Predictors of death

## Abstract

**Objective:**

To determine the sociodemographic characteristics, risk factors, treatment outcomes and predictors of death of newly diagnosed patients with venous thromboembolism (VTE).

**Design:**

A retrospective study

**Settings:**

University of Ghana Medical Centre (UGMC) Ltd, a 650-bed quaternary hospital located in Accra, Ghana.

**Participants:**

One hundred and eighty-six adult patients admitted with a diagnosis of deep vein thrombosis and pulmonary embolism (PE), confirmed by Computed tomography pulmonary angiography (CTPA), from January 2022 to April 2024, were included in this study. All patients with a differential diagnosis of DVT or PE for whom confirmation by Doppler USG and CTPA could not be obtained were excluded.

**Main outcome measure:**

Risk factors, treatment outcomes and predictors of death of patients diagnosed with venous thromboembolism

**Results:**

The mean age of study participants was 62.7 years (±15.5), and a slightly higher proportion were female (n=108, 58.1%). The mortality rate was 16.3%. Close to half of the patients (n=88, 47.3%) had a history of immobilisation as the predominant risk factor for VTE. On multivariate analysis, increasing age (OR 1.04, 95% CI 1.00-1.08), increasing urea level (OR 1.12, 95% CI 1.03 – 1.21) and presence of cancer (OR 6.02, 95% CI 0.003) remained significant predictors of mortality.

**Conclusion:**

Immobilisation was the main risk factor for VTE in this study. In-hospital mortality was relatively high, with death in 1 in 6 patients diagnosed. Patients with advanced age, high urea and malignancy should be monitored closely and early aggressive treatment instituted to reduce mortality.

**Funding:**

None declared

## Introduction

Venous thromboembolism (VTE) comprises two interconnected conditions within the same disease spectrum: deep vein thrombosis (DVT) and pulmonary embolism (PE). About 60–70% of patients with symptomatic PE have underlying DVT.^1^ Globally, VTE is the third most common acute cardiovascular disease, after stroke and coronary artery disease.^2^ VTE affect close to 10 million people every year worldwide and causes an enormous financial burden on patients, health facilities, and the state.^3^,^4^ The burden of VTE in Africa is enormous, with 1 out of 2 inpatients considered at risk of VTE. In a multicentre study across 5 African countries, Kingue and colleagues reported that the risk of VTE among surgical patients was 43.8%, whereas among medical patients it was 62.3%.^5^ PE is fatal in about one-third of cases, with a failure to diagnose ante mortem in roughly 67% of cases.^6^ Approximately 34% of PE occur rapidly.^6^ A high proportion of clinically unsuspected VTE leads to significant diagnostic and therapeutic delays, and this accounts for substantial morbidity and mortality.^6^

VTE risk factors can be divided into patient-related factors, disease states, hematologic disorders, and surgical factors, and risk is additive. Patient-related factors include age older than 40 years, immobility, obesity, varicose veins and the use of medications such as oestrogen. Disease conditions such as cancer, nephrotic syndrome, congestive heart failure, inflammatory bowel disease, recent myocardial infarction, stroke or spinal cord injury with paralysis, and long-bone fracture confer increased risk of VTE.^7^

Furthermore, duration and type of surgical procedure are related to surgical factors which increase the risk of VTE. Proximal DVT occurred in 50% of patients who had undergone hip surgery, with twisting of the femoral vein during total hip replacement thought to account for this. For patients who have undergone knee surgery, the incidence of DVT tends to be higher.^7^ Previous DVT, bed rest, cancer diagnosis, and peripherally inserted central venous catheters have been identified as highly predictive of VTE among hospitalised medical patients. 8

The risk of VTE in patients in Africa ranges between 31.7 to 75% and between 34.2% and 96.7% of these received prophylaxis.^9^ Thromboprophylaxis has been documented to be effective and has been found to reduce the rate of DVT and fatal PE by 30 to 65%.^10^, ^11^ This can be achieved by mechanical (external compression and early ambulation) and pharmacologic means (heparin, warfarin, direct oral anticoagulants, aspirin, etc). The main clinical presentation of VTE includes complaints related to either DVT, such as lower extremity swelling, warmth to touch and tenderness, or those associated with PE. Signs and symptoms of PE can include acute onset of difficulty in breathing- often the commonest symptom, pleuritic chest pain, cough and haemoptysis (especially in relation to smaller PE near the pleura), syncope (linked to massive PE), tachypnoea, tachycardia, fever, cyanosis, accentuated second heart sound, and a sense of impending doom with apprehension and anxiety.^6^ Management options include the use of anticoagulant medications such as low or high-molecular-weight heparin, administration of an oral coumarin derivative such as warfarin, or the use of oral factor Xa inhibitors such as rivaroxaban. Thrombolytic therapy, including tissue plasminogen activator such as recombinant agents alteplase, reteplase, and tenecteplase, is used for the initial management of patients presenting with acute high-risk PE. Surgical interventions available include thrombectomy, embolectomy (limited to massive PE when thrombolysis is contraindicated or other treatments have failed) and venous interruption (currently rare).

Data from Ghana on VTE is scarce, and therefore, the gravity of VTE is likely to be underestimated. Few studies have been carried out on VTE burden in Ghana.^12^,^13^,^14^ There is a dearth of knowledge about risk factors, clinical presentation, in-hospital mortality and the predictors of death of VTE patients in Ghana. This study aimed to assess the baseline characteristics, risk factors, treatment outcomes and predictors of death amongst patients with VTE in a tertiary hospital in Ghana.

## Methods

### Study design and study population

This was a retrospective cross-sectional study in which data were retrieved from the hospital's electronic patient folder for the period 1^st^ May 2024 to 14^th^ May 2024 at the University of Ghana Medical Centre (UGMC) Ltd, a 650-bed quaternary hospital located in Accra, Ghana. All adult patients admitted to male and female medical wards and to the emergency room were eligible for inclusion in the study. Adult patients admitted with a diagnosis of DVT, confirmed by Doppler ultrasound, and PE, confirmed by Computed tomography pulmonary angiography (CTPA), from January 2022 to April 2024 were included in this study. All patients with a differential diagnosis of DVT or PE for whom confirmation by Doppler USG and CTPA could not be obtained were excluded.

Information extracted from the folder included the following: age, sex, clinical presentation including pulse and blood pressure, risk factors (such as cancer, immobilisation, surgery, previous DVT or PE, cardiovascular risk factors, etc), treatment given and outcome of admission (discharge or death). The following laboratory values were also obtained from the records: haemoglobin (Hb), white blood cells (WBC), platelets (PLT), creatinine (Cr), urea, total bilirubin (T. bil.), and albumin. The duration of hospital admission and the medications received were also recorded.

### Ethical approval

The study was approved by the University of Ghana Medical Centre Institutional Review Board (UGMC/IRBREVIEW/064/24). All data collected were secondary data from a review of archived medical records and were de-identified and anonymised prior to analysis. Informed consent was not required and was waived by the ethical review board.

### Statistical Analysis

Descriptive statistics were used for patient characteristics, including age and sex, with means and standard deviations, or medians with interquartile ranges, reported for continuous variables. Frequencies (percentages) were used to describe categorical variables. The chi-square test and analysis of variance (ANOVA) were used to compare the differences between groups. Binary logistic regression was used to identify factors associated with mortality. In the logistic regression model, death (yes/no) was modelled as the outcome, and predictor variables included age (continuous), sex (male/female), and clinical findings (systolic and diastolic blood pressure, pulse, respiratory rate, comorbidity, cancer, etc.). In the multivariable model, factors identified as significantly associated with mortality in the univariate analysis (p<0.05) were included, along with age and sex, to estimate the odds ratio (OR). A significance level of 0.05 was used. All tests were two-sided with a 95% confidence interval (p-value 0.05). Data analysis was performed using Stata, version 17, Stata Corp software.

## Results

### Demographic and clinical characteristics of patients with VTE

In total, 186 patients were enrolled on this study. The mean age of study participants was 62.7 years (±15.5) and a slightly higher proportion were female (n=108, 58.1%) (Table 1).

**Table 1:** Demographic and clinical characteristics of patients with DVT and PE

	All patients n=186	DVT n=43	PE n=108	PE+DVT n=35	p value*
**Age, years, Median, (IQR)**	65(53-72)	67 (58-77)	64 (52–71.5)	65 (47-71)	0.17
**Age, years, Mean (±SD)**	62.7, 15.5	66.6, 15.5	61.5, 15.2	61.5, 16.3	0.18
**Sex, n (%)**					
**Male**	78 (41.9)	24 (55.8)	43 (39.8)	11 (31.4)	0.08
**Female**	108 (58.1)	19 (44.2)	65 (60.2)	24 (68.6)	
**Blood Pressure (mmHg), Mean (±SD)**					
**Systolic**	121.0 (27.0)	125.3 (21.7)	121.0 (27.3)	115.6 (31.0)	0.28
**Diastolic**	74.8 (19.8)	71.3 (15.6)	76.7 (19.9)	73.1 (23.4)	0.28
**Pulse (bpm), Mean (±SD)**	97 (17)	89 (16)	100 (17)	94(18)	**0.001**
**Spo2 (%), Mean (±SD)**	91 (10)	95 (12)	90 (9)	91 (7)	**0.02**
**Respiratory rate (cpm) Mean, (±SD)**	24 (7)	20 (4)	25 (6)	25 (11)	**0.001**
**Haemoglobin g/dl, Mean (±SD)**	12.0 (3.7)	12.0 (6.5)	12.4 (2.0)	10.7(2.5)	0.06
**WBC x10^9^/l, Mean (±SD)**	9.9 (14.3)	9.0 (5.5)	11.0 (18.3)	7.9 (3.4)	0.47
**Platelet x10^9^/l, Mean (±SD)**	233.8 (104.0)	231(120)	227 (99)	255 (98)	0.47
**Urea, mmol/L, Mean (±SD)**	7.2 (5.5)	7.14 (5.7)	6.8 (4.9)	8.7 (7.1)	0.20
**Creatinine, umol/L Mean, (±SD)**	129.6 (166.2)	130.1(120.8)	128.2 (200.3)	133.0 (76.8)	0.99
**Albumin, g/L, Mean (±SD)**	35.8 (8.7)	35.9 (8.5)	35.1 (8.6)	35.3 (9.2)	0.95
**Total Bilirubin, umol/L, Mean (±SD)**	14.6 (33.1)	10.4 (8.6)	17.7 (42.3)	9.9 (7.1)	0.41
**Co-morbidity present, n (%)**	101 (54.3)	25 (58.1)	55 (50.9)	21 (60.0)	0.546
**Duration of admission, days, median (IQR)**	5 (3-9)	5 (3-10)	5 (3-9)	5 (3-8)	0.69
**Outcome of admission, n (%)**					
**Discharge**	149 (83.7)	34 (82.9)	92 (87.6)	23 (71.88)	0.11
**Death**	29 (16.3)	7 (17.1)	13 (12.4)	9 (28.2)	

CPM-cycles per minute; BPM-beats per minute; WBC-White blood cell

* Anova or Chi-square test

More than half of all VTE patients (54.3%) had a co-morbidity, with the most common co-morbid conditions being hypertension (55.5%) and Type 2 Diabetes (32.8%) (Supplemental Table 1). The median duration of hospital admission for patients with VTE was 5 days (IQR 3-9). The mortality rate was 16.3% among patients with a single diagnosis of either PE or DVT, and 28.2% in those with a dual diagnosis of both PE and DVT.

### Presenting symptoms, risk factors, and management of VTE patients

**Figure 1:**
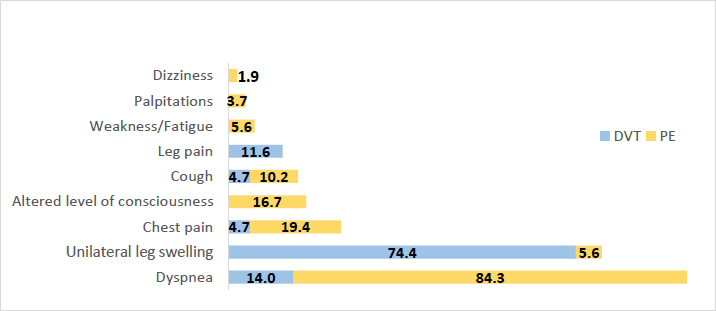
Percentage distribution of symptoms in patients diagnosed with DVT and PE

A high proportion of patients diagnosed with PE presented with dyspnoea (84.3%), and a smaller proportion presented with chest pain (19.4%) and altered level of consciousness (16.7%) (Figure 1).

**Figure 2:**
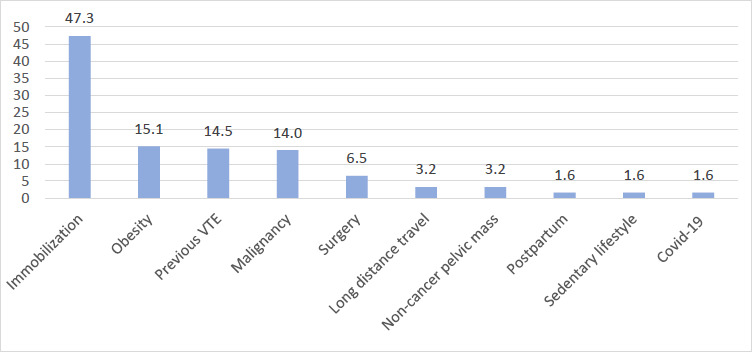
Percentage distribution of risk factors for patients diagnosed with VTE

**Figure 3:**
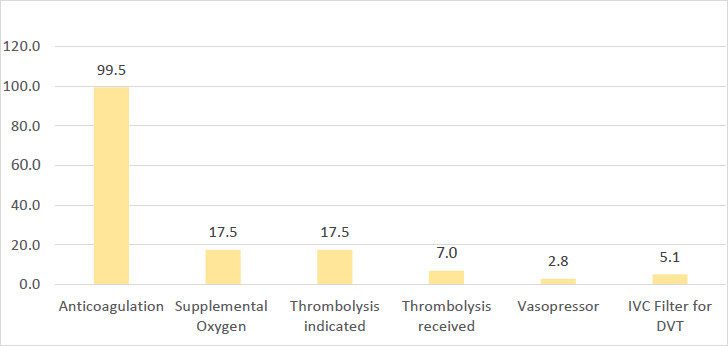
Treatment undertaken by patients diagnosed with VTE

For patients with DVT, the majority presented with unilateral leg swelling (74.4%) and leg pain (11.6%). Close to half of the patients (n=88, 47.3%) had a history of immobilisation as the predominant risk factor for VTE (Figure 2). Other commonly reported risk factors included obesity (n=28, 15.1%), previous VTE (n=27, 14.5%) and malignancy (n=26, 14.0%). The most common types of malignancy among patients managed on the medical ward for VTE were prostate (n=6), unknown primary (n=6), breast (n=3) and pancreatic (n=3) cancer (Supplemental Table 2). In this study, thrombolysis was indicated for 25 out of 143 (17.3%) patients with PE (Figure 3), and less than one third of those in whom thrombolysis was indicated (n=7, 28%) undertook the procedure.

### Predictors of in-hospital mortality among VTE patients

On univariate analysis, factors associated with mortality among VTE patients included increasing age (OR 1.04 95% CI), increasing urea (OR 1.15, 95% CI 1.08-1.23), presence of co-morbidity (OR 4.86, 95% CI 1.76-13.43) and presence of cancer (OR 11.71, 95% CI 4.52-30.36) (Table 2). On multivariable analysis, increasing age (OR 1.04, 95% CI 1.00-1.08), increasing urea level (OR 1.12, 95% CI 1.03 – 1.21) and presence of cancer (OR 6.02, 95% CI 0.003) remained significant predictors of mortality among patients with VTE (Table 3).

**Table 2:** Univariate analysis of factors associated with mortality in VTE patients

	OR	95% CI	p value
**Age**	1.04	1.01-1.07	**0.012**
**Sex (Female)**	1.72	0.73-4.02	0.212
**Duration of admission**	1.02	0.97-1.08	0.38
**Systolic**	0.98	0.96-0.99	**0.005**
**Diastolic**	0.97	0.95-0.99	**0.021**
**Pulse**	1.03	1.01-1.06	**0.013**
**SPO2**	0.98	0.95-1.02	0.340
**Urea**	1.15	1.08-1.23	**0.000**
**Creatinine**	1.00	0.99-1.00	0.153
**Hb**	0.77	0.65-0.92	**0.004**
**WBC**	1.00	0.98-1.02	0.821
**Co-morbidity**	4.86	1.76-13.43	**0.002**
**Cancer**	11.71	4.52-30.36	**0.000**
**Thrombolysis**	0.63	0.08-5.23	0.668
**Vasopressor**	5.44	0.73 – 40.33	0.097

**Table 3:** Multivariate analysis of factors associated with mortality in VTE patients

	OR	95% CI	p value
**Age (years)**	1.04	1.00-1.08	**0.041**
**Sex (Female)**	2.18	0.68-7.06	0.192
**Systolic BP**	0.97	0.93-1.00	0.071
**Diastolic BP**	1.04	0.99-1.09	0.087
**Pulse bpm**	1.01	0.98-1.05	0.336
**Haemoglobin g/dl**	0.96	0.84-1.09	0.497
**Urea mmol/l**	1.12	1.03-1.21	**0.008**
**Cancer (yes)**	6.02	1.82-19.89	**0.003**
**Comorbidity (yes)**	2.92	0.80-10.61	0.104

## Discussion

VTE is the third most common acute cardiovascular condition, following stroke and coronary artery disease. Despite the significant burden of VTE demonstrated globally, epidemiological data on this disease from Ghana is lacking. This study aimed to determine the sociodemographic characteristics, risk factors, treatment outcomes, and predictors of death in newly diagnosed patients with VTE at a referral hospital in Ghana. The mean age of study participants was 62.7 years (±15.5). The median hospital admission duration was 5 days. The mortality rate was 16.3%. A high proportion of patients diagnosed with pulmonary embolism presented with dyspnoea, and for patients with deep vein thrombosis, the majority presented with unilateral leg swelling. Close to half of the patients had a history of immobilisation as the predominant risk factor for VTE. On multivariate analysis, increasing age, increasing urea level and presence of cancer were significant predictors of mortality.

The mean age of hospital patients admitted with VTE in this study was 62.7 ± 15.5. This is similar to the age group reported in a study by Mok and colleagues^15^ among Asians, but higher than that reported in studies conducted in other African and Western countries.^12^, ^16^ The differences between the age groups may be attributable to participant characteristics, such as risk factors and inpatient versus outpatient status. Mok et al.'s study included participants with acute PE only; Osei et al. conducted their study among deceased patients; and the study conducted in Cameroon included inpatients only.^12^, ^15^, ^16^

Hypertension, diabetes, malignancy, stroke, congestive heart failure, chronic kidney disease, osteoarthritis and pneumonia were common comorbidities associated with (Table 1). These comorbid conditions, such as stroke and osteoarthritis, lead to venous stasis due to immobilisation, malignancy leads to a hypercoagulable state and hypertension, diabetes, for instance, leads to vascular endothelial injury. Venous stasis, hypercoagulable state and vascular endothelial injury are the triad of factors that constitute the pathogenic condition of VTE, first described in 1856 by Virchow's Triad. This is similar to studies published by Lee et al.,^17^ and Huerta et al.,^18^ where these were the predominant comorbidities among VTE patients. Consistent with other studies worldwide, patients in this study exhibited a wide range of clinical features.

The most common presentations included dyspnoea, observed in about 84.3% of patients diagnosed with PE, and unilateral leg swelling, seen in 74.4% of patients with DVT. Other reported symptoms included chest pain, altered consciousness, cough, leg pain, fatigue, palpitations, and dizziness. VTE should be considered as a likely diagnosis in patients presenting with these symptoms, particularly dyspnoea and unilateral leg swelling. Immobilisation, obesity, previous VTE and malignancy were the main risk factors of VTE in this study. These risk factors are similar to those reported in studies from Africa and other regions worldwide.^15^,^19^, ^20^

The in-hospital mortality rate of patients in this study was 16.3%. This is comparable to data reported by Kengue et al.^21^ and Ngahane et al.^19^ In contrast, a lower mortality rate of 7.0% was recorded by Bakebe et al.^22^ in Kinshasa, 1.1% by a study published by Pollack et al.^23^ in the U.S. S. and Monk et al.^15^ reported a 5% mortality rate in a study conducted in Asia. However, higher mortality rates were reported by Abah et al. 16 in Cameroon (47.6%) and by Amar et al.24 in Tunisia (40%).

These differences in mortality rates may be due to variations in VTE severity, underlying risk factors, comorbidities, and the resources available for managing these patients. Additionally, the type of VTE involved in the study—whether PE, DVT, or both could also account for these differences. The management setting, whether in the ward or the emergency room, may also account for differences in mortality rates observed across studies. The length of stay in the emergency department is usually lower than in the conventional medical ward.

In this study, multivariable analysis identified age, high urea, and cancer as statistically significant predictors of mortality. Multiple studies have demonstrated that advanced age correlates with unfavourable outcomes in patients with VTE.^17^, ^25^ This adverse outcome linked to ageing is likely due to the numerous comorbidities common in the elderly population. Additionally, patients with cancer who develop VTE have a reduced life expectancy. Research indicates that patients with cancer have a 4- to 8-fold increased risk of mortality following an acute VTE event compared to those without cancer.^26^ In a large population-based study by Serensen and colleagues, the one-year survival rate for patients with cancer and VTE was 22%, compared to 36% for control patients without VTE, matched for cancer type, sex, age, and year of diagnosis.^27^ This elevated mortality rate may be attributed to both thromboembolism and the more aggressive progression of cancers associated with VTE. According to a study by Huerta et al.^18^ in the UK, cancer presents a greater relative risk of fatal pulmonary embolism (PE) compared to nonfatal PE in patients without cancer. High urea is associated with an increased risk of venous thromboembolism (VTE) in patients with acute ischemic stroke (AIS).^28^ Urea is a common laboratory test used to evaluate prerenal azotaemia, acute tubular necrosis, and dehydration. Given the established link between VTE and dehydration following AIS, 28 our findings suggest that encouraging fluid replacement may reduce the likelihood of death from VTE, particularly in dehydrated patients with high urea levels.^28^ Our study thus offers a new perspective on predicting mortality in VTE patients with high urea.

Although the present study is among the first to report on the clinical characteristics, risk factors, and treatment outcomes of VTE patients in Ghana, it has several limitations. It is retrospective and was conducted at a single site, which is not representative of the entire country of Ghana. Additionally, this was among medical and emergency room patients; surgical, obstetric, and gynaecology patients were excluded.

## Conclusion

The mean age of study participants was 62.7 years (±15.5), and a slightly higher proportion were female. Immobilisation, obesity, previous VTE and cancer were the main risk factors for VTE in this study. In-hospital mortality was relatively high, with death occurring in 1 in 6 patients diagnosed with VTE. Patients with advanced age, high urea and a history of malignancy should be monitored closely and early aggressive treatment instituted to reduce mortality. The prognosis should be discussed with patients and their relatives, taking into account predictors of mortality. Further studies from multiple sites, with larger sample sizes, are needed to provide a detailed description of the Ghanaian context, with a focus on characterising risk factors and treatment outcomes.

## Supplement Table 1

**Table:**

Co-morbidity	All	DVT	PE	Both	p value
**Hypertension**	**101 (55.5)**	**20 (46.5)**	**62 (58.0)**	**19 (54.3)**	**0.445**
**Diabetes**	**61 (32.8)**	**19 (44.2)**	**32 (29.6)**	**10 (28.6)**	**0.191**
**Malignancy**	**26 (14.0)**	**11 (25.9)**	**10 (9.3)**	**5 (14.3)**	**0.033**
**Stroke**	**13 (7.0)**	**3 (7.0)**	**6 (5.6)**	**4 (11.3)**	**0.496**
**CCF**	**9 (4.8)**	**0 (0)**	**6 (5.6)**	**3 (8.6)**	**0.186**
**CKD**	**4 (2.2)**	**1 (2.3)**	**2 (1.9)**	**1 (2.9)**	**0.932**
**Pneumonia**	**6 (3.2)**	**0 (0)**	**5 (5.4)**	**1 (2.9)**	**0.345**
**Osteoarthriti**	**7 (3.7)**	**2 (4.7)**	**3 (2.8)**	**2 (5.7)**	**0.687**

## Supplement Table 2

**Table:**

Cancer Location	Frequency	Percent	Cum.
**Brain**	1	3.85	3.85
**Oesophagus**	1	3.85	7.69
**Nasopharyngeal**	1	3.85	11.54
**Breast**	3	11.54	23.08
**Liver**	1	3.85	26.92
**Gallbladder**	1	3.85	30.77
**Pancreas**	3	11.54	42.31
**Endometrium**	1	3.85	46.15
**Prostate**	6	23.08	69.23
**Rectum**	1	3.85	73.08
**Multiple Myeloma**	1	3.85	76.92
**Primary Unknown**	6	23.08	100
**Total**	26	100	
